# Fabrication of Microspheres from High-Viscosity Bioink Using a Novel Microfluidic-Based 3D Bioprinting Nozzle

**DOI:** 10.3390/mi11070681

**Published:** 2020-07-14

**Authors:** Shanguo Zhang, Guiling Li, Jia Man, Song Zhang, Jianyong Li, Jianfeng Li, Donghai Li

**Affiliations:** 1 Key Laboratory of High Efficiency and Clean Mechanical Manufacture of MOE, School of Mechanical Engineering, Shandong University, Jinan 250061, China; zsggg@mail.sdu.edu.cn (S.Z.); zhangsong@sdu.edu.cn (S.Z.); ljy@sdu.edu.cn (J.L.); ljf@sdu.edu.cn (J.L.); 2 Key National Demonstration Center for Experimental Mechanical Engineering Education, Shandong University, Jinan 250061, China; 3 School of Medicine, Tsinghua University, Beijing 100084, China; lgl@mail.tsinghua.edu.cn; 4 Advanced Medical Research Institute, Shandong University, Jinan 250012, China

**Keywords:** droplet-based bioprinting, microfluidic system, phase-inversion method, microcomponent

## Abstract

Three-dimensional (3D) bioprinting is a novel technology utilizing biocompatible materials, cells, drugs, etc. as basic microcomponents to form 3D artificial structures and is believed as a promising method for regenerative medicine. Droplet-based bioprinting can precisely generate microspheres and manipulate them into organized structures with high fidelity. Biocompatible hydrogels are usually used as bioinks in 3D bioprinting, however, the viscosity of the bioink could be increased due to the additives such as cells, drugs, nutrient factors and other functional polymers in some particular applications, making it difficult to form monodispersed microspheres from high-viscosity bioink at the orifice of the nozzle. In this work, we reported a novel microfluidic-based printing nozzle to prepare monodispersed microspheres from high-viscosity bioink using the phase-inversion method. Different flowing conditions can be achieved by changing the flow rates of the fluids to form monodispersed solid and hollow microspheres using the same nozzle. The diameter of the microspheres can be tuned by changing the flow rate ratio and the size distribution of the microspheres is narrow. The prepared calcium alginate microspheres could also act as micro-carriers in drug delivery.

## 1. Introduction

Nowadays, millions of people are waiting for organ transplantation due to organ damage, but the number of organ donations is far from meeting the needs of patients, and organ shortage has become a serious crisis in the field of public health [1,2]. The organ substitutes made from tissue engineering are playing an increasingly important role in solving these problems [3,4]. Three-dimensional (3D) bioprinting technology involves the intersection of life sciences, computers, materials science and other disciplines. It can precisely place different types of cells and materials in predetermined positions to print composite structures [5].

There are three different types in bioprinting fields, including laser-based [6,7,8], extrusion-based [9,10,11] and droplet-based bioprinting [12,13]. Laser-based bioprinting technology is developed from the Laser Induced Forward Transfer (LIFT) technique. When a pulsed laser is focused on the laser absorber through the transparent substrate, the laser absorber absorbs most of the heat. A small amount of heat is absorbed by the biomaterial film near the laser absorbing layer and a vapor bubble is formed. The vapor bubble expands and deforms at the junction of the biofilm and air, thus forcing a very small number of biomaterials to leave the donor glass and deposit on the receiving glass [14]. As a nozzle-free technology, laser-based bioprinting can effectively solve the problems in the conventional printing methods, such as the easy blocked pinhole or nozzle. Moreover, it is also a non-contact printing method, which can deposit different kinds of biomaterials on multiple target plates without backfilling and cleaning the target plates repeatedly. However, laser-based bioprinting is limited because of the high cost and difficulties in constructing well-defined 3D architectures [15], and it cannot be used to print artificial tissue.

The extrusion-based bioprinting technique is a combination of a fluid-dispensing system and an automated robotic system for extrusion and bioprinting, respectively. The liquid or sol bioinks in the nozzle cavity are extruded to form microfibers as the structural construction unit through the nozzle. After the formation of microfiber, the computer-controlled the nozzle movement for the two-dimensional deposition of the microfibers, and the three-dimensional deposition effect is realized by layer switching from bottom to top [16]. Extrusion-based bioprinting has a fast printing speed which is more suitable for building structures with large sizes. Generally, the minimum feature size is usually beyond 100 μm [17]. Moreover, the shear stress produced in the nozzle tip will cause great damages to cells [18]. Thus, it can be deficient when applied to the high-resolution patterning of multiple cell types.

Taking the droplet as the basic unit of bioprinting, different types of printing structures with the high-resolution combination can be realized. Comparing with the former two methods, the droplet-based bioprinting has the advantages of fast printing speed, low cost, small volume droplets and the great control over the deposition pattern [19]. In droplet-based bioprinting, the hydrogel has received increasing attention as a component of the bioink. The viscosity of the bioink plays an important role in 3D bioprinting. For example, the hydrogel bioink should have enough viscosity, which means the 3D component could maintain its structure before being solidified. Moreover, in many situations, cells, polymers or other additives are usually added into the bioink according to the application, inevitably leading to the increase of viscosity. High-viscosity hydrogels for bioprinting can protect cells from damage during printing and achieve accurate deposition [12]. However, high-viscosity hydrogels with a poor fluidity are easy to plug the print nozzle, and only fiber-based structure could be formed from high-viscosity bioink, instead of microspheres. Paxton et al. [20] used the high-viscosity alginate solution as the bioink to print 3D structures. The result showed that an 8% *w/v* alginate solution could retain the unit of fibers and solidify the layers by additional crosslinking methods (CaCl_2_). Some certain microcomponents, such as microspheres, are difficult to make from a high-viscosity bioink. Thus, the research on the bioprinting technology using high-viscosity hydrogels is necessary.

Most biocompatible hydrogels have the property of thermal responsiveness, which is characterized that the viscosity of the hydrogels will decrease as the temperature increases. Compound hydrogels of alginate and gelatin are usually utilized as bioinks for bioprinting. After heating in the printing channel, the composite hydrogels with high-viscosity will turn into low-viscous hydrogels. When the low-viscosity hydrogels touch with the cooling plate through the printing nozzle, the low-viscous hydrogels will transform into high-viscosity hydrogels again. The composite hydrogels with high viscosity will quickly solidify to form a stable 3D structure during deposition [21,22,23]. However, the essence of the researches is to meet the general requirements of bioprinting by adjusting the compatibility or viscosity of the bioink instead of improving the bioprinting technology of high-viscosity hydrogels. Moreover, it is easy to cause damage to the living cells encapsulated by the hydrogels in the process of heating.

**Figure 1:**
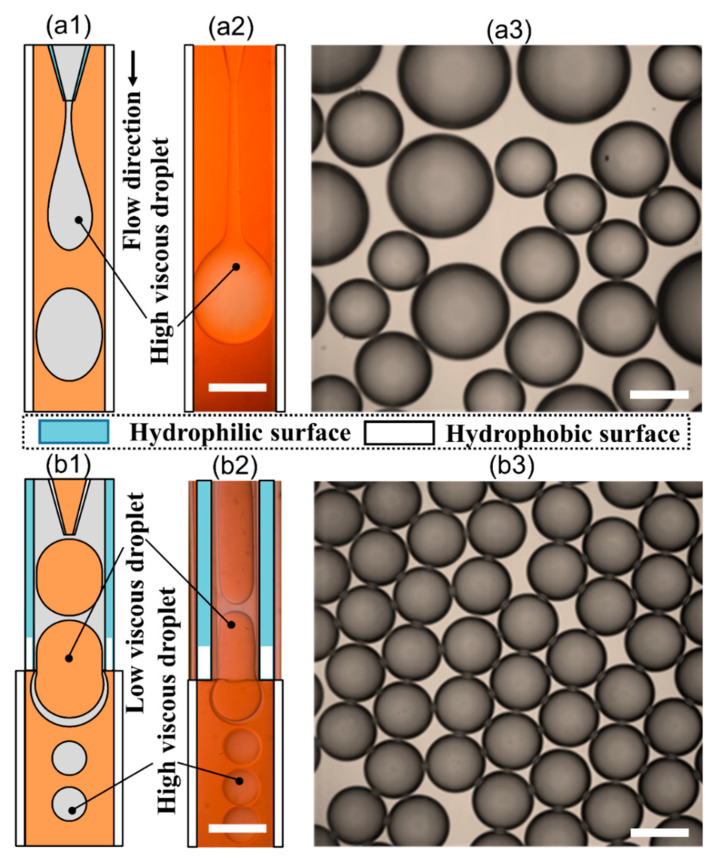
High-viscosity fluid in the microfluidic chip. (**a1**) Schematic illustration of jetting mode when preparing high-viscosity microspheres using high-viscosity fluid as the inner phase; (**a2**) Jetting mode inside the microchannel captured in high-speed camera; (**a3**) Polydispersed high-viscosity microspheres prepared from a jetting mode; (**b1**) Schematic illustration of dripping mode when preparing high-viscosity microspheres using the phase-inversion method; (**b2**) Dripping mode inside the microchannel captured in a high-speed camera; (**b3**) monodispersed high-viscosity microspheres prepared from the phase-inversion method. All scale bars are 500 μm.

Recently, microfluidic technology began to be used as a droplet-based bioprinting method [24,25,26]. The accurate control of fluid and the precise structure of microchannel make it possible to prepare microdroplets with uniform size distribution and realize the accurate printing of 3D structure. However, it has the same difficulties of making monodispersed microspheres from high-viscosity bioink in a microfluidic device, as shown in Figure 1(a1–a3). As shown in Table 1, the existing droplet-based technologies are limited in printing high-viscosity fluids.

Alginate is extracted from various algae including *Macrocystis*, *Laminaria*, *Ascophyllum*, *Alario*, *Ecklonia*, *Eisenia*, *Nercocystis*, *Sargassum*, *Cystoseira* and *Fucus*, indicating that the raw sources of alginate are abundant [27]. It is hard to degrade in mammals because of the lack of an enzyme, alginate lyase (ALGL), which can break the chain of alginate polymers. However, calcium alginate can degrade in the human body, which can be explained by the exchange of calcium ions in colloids and sodium ions in body fluids [28,29]. As a kind of cell immobilization and culturing matrix, calcium alginate has an obvious promoting effect on cell growth [30]. Thus, calcium alginate hydrogel is a kind of biocompatible and biodegradable material and exhibits hydrophilicity with interconnected porous structures, which can provide a stable space for drug storage and cell proliferation while maintaining cell function [31]. Calcium alginate hydrogel has been given priority in the development of drug delivery systems due to properties such as swelling ability, mucosal adhesion and sol/gel conversion ability [32,33].

The integration of nanotechnology and modern medicine has made great progress. In particular, magnetic nanomaterials, with their excellent magnetic properties and good biocompatibility, have become hot functional materials [34,35]. Magnetic nanoparticles can not only be directionally transported to target areas using external magnetic fields but generate thermal effects under the action of alternating magnetic fields to induce structural changes of heat-sensitive materials, thus controlling the release of drugs [36]. However, ultra microsized non/low-toxic materials have the possibility of turning into toxic substances with incorrect dosage. Ultra-microparticles with an average diameter of 20 nm are more likely to cause inflammation in vivo than the particles with an average diameter of 250 nm. Overdosed nanoparticles can also cause severe epithelial cell proliferation, pulmonary inflammation, pulmonary fibrosis and even lung tumors [37,38]. Thus, it is important to control the concentration of nanoparticles and 50 μg/mL may be a safe parameter [39]. Compared with different nano-iron oxides, it can be seen that nano–Fe_3_O_4_ particles have stronger magnetic and superparamagnetic properties, which can achieve targeted positioning efficiently. Moreover, nano–Fe_3_O_4_ particles have the advantages of large surface-to-volume ratio, good affinity, no biotoxic side effect and can be eliminated with the metabolite after exerting the drug effect.

Magnetic drug delivery microsphere is a new targeted drug delivery system and has been studied extensively in recent years [40,41,42,43]. Magnetic drug microspheres are mainly composed of magnetic materials, biocompatible materials and drugs. The targeting mechanism is to fabricate magnetic drug-loaded microspheres by dispersing drugs and appropriate magnetic components in biocompatible materials. Then, it is introduced into the body through intravenous or arterial injection. Under the effect of the external magnetic field, the magnetic microspheres loaded with drugs are concentrated and located in the target focal zone, improving the concentration of drugs in the target area [44,45]. According to the properties of the magnetic drug-loaded microspheres, we can realize the controlled and predictable rate of drug release with smaller doses of the drug and the avoidance of acute toxicity directed against endothelium and normal parenchyma cells [46]. For example, Chen made a magnetic drug delivery system by chemically binding drugs to nano–Fe_3_O_4_ particles and encapsulated them in the shell of functionalized porous SiO_2_ particles. The porous SiO_2_ shell could prevent the loss of drugs and magnetic nanoparticles before they reached the target area and decrease the release rate of drugs [47]. Meyers injected the tiny iron particles intravenously into the dog’s leg vein and used a large magnet to externally guide them, achieving a successful lymphoid targeting effect [48].

In this study, we reported a novel method to prepare monodispersed calcium alginate microspheres for bioprinting directly from the high-viscosity fluid with 1440 mPa·s using a phase-inversion method. The oil-in-alginate droplets can transfer into alginate-in-oil droplets spontaneously simply through changing the wettability of the microchannels, as shown in Figure 1(b1,b2). Then we studied the parameters that influence the formation of high-viscosity calcium alginate microspheres. It was found that the microspheres prepared in the phase-inversion method exhibited high monodispersity and sphericity. Additionally, a drug release experiment in a model vessel was employed to show the potentials of calcium alginate microspheres with magnets in the controlled drug-release property. For further research on organ printing, the pluripotent stem cells and the hydrogels matrix should extract from human tissue. Then, the differentiated cells will be cultured in hydrogels to form the bioinks needed for 3D printing. The 3D model of the organ with blood vessels should be established by the computer. Based on the organ model, the organ can be fabricated using the droplet-based bioprinting technology with the bioinks [49]. However, the viscosity of bioink in this process is not always low enough for bioprinting. The technique introduced in this article as one step in organ bioprinting mainly focused on the fabrication of microspheres from high-viscosity bioink, which could promote the development of organ bioprinting.

**Table 1:** Maximal viscosity required by different droplet-based bioprinting technologies.

Droplet-Based Bioprinting Technology	Schematic Drawing of Different Bioprinting Systems	Maximal Viscosity (mPa·s)	Ref.
Acoustic bioprinting	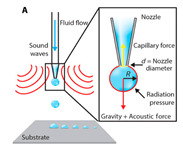	200	[50,51]
Inkjet bioprinting	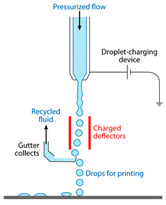	10	[52,53]
Microvalve bioprinting	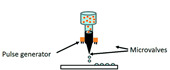	200	[54,55]
Microfluidic bioprinting	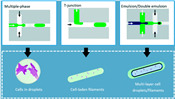	10	[56,57,58]

## 2. Materials and Methods

### 2.1. Materials

Sodium alginate (Sigma, *M*_w_ = 240,000 g/mol) was added into deionized water (DI) water to achieve a concentration of 0.5 wt%. Calcium chloride (Sinopharm Chemical Reagent) was added into DI water to achieve 0.1 wt%. Sodium alginate solution and calcium chloride solution were mixed to achieve the volume ratio 4:1, used as high-viscosity fluid (≈1440 mPa·s) in a microfluidic experiment. Into the above mixture, 1 wt% Fe_3_O_4_ nanoparticles (50–300 nm, Aladdin Co., Ltd. Shanghai, China) was added to make magnet-controlled microspheres. Paraffin oil (Sinopharm Chemical Reagent) with 1 wt% Span 80 was used as low viscous fluid (≈29 mPa·s) and was dyed in orange color. Octadecyltrichlorosilane (OTS, Sigma-Aldrich, St. louis, MO, USA) was used to treat the capillary tube to be hydrophobic, while triethoxysilane (TTS, Sigma-Aldrich, St. louis, MO, USA) was used to treat the capillary tube to be hydrophilic. Blue-O was used as a model drug and phosphate buffer saline (PBS) solution was used as a model human body fluid in the drug release experiment.

### 2.2. Microfluidic Chip Setup

The device has a co-flow structure, where the injection glass tube has inner diameter (I.D.) = 100 μm and outer diameter (O.D.) = 200 μm while the collection tube has I.D. = 580 μm and O.D. = 1.0 mm. For the generation of aqueous droplets, e.g., the device in Figure 1(b1), we immersed the exit of the collection tube in the OTS solution for 0.1 mm. After that, the OTS solution would flow into the glass tube due to the capillary force. We would stop the immersing process and input nitrogen from the inlet of the collection tube for 1 min when the OTS solution flowed for 0.5 mm. Then, the length of 0.5 mm of the exit of the collection tube would be hydrophobic; for the generation of non-aqueous droplets, the upstream part of the collection tube is treated with OTS to be hydrophobic, while the length of 0.5 mm of the exit is treated with TTS to be hydrophilic. After that, the treated exit of the collection tube is inserted into a hydrophobic square tube with I.D. = 1.05 mm totally treated by the OTS solution and nitrogen blow, and the interstices between the collection tube and the square tube are sealed with epoxy glue. The low-in-high-viscosity segment flow is formed with the co-flowing structure and then is broken up at the step-down edge of the exit as illustrated in Figure 1(b2).

### 2.3. Preparation of Microspheres

First, we treated a capillary tube with a partial hydrophilic section and the partially hydrophobic section as a collection tube and inserted a conical tube to the collection tube to make a co-flow microfluidic chip. Second, we flew high-viscosity alginate fluid and low viscous oil phase fluid into the chip as the outer phase and inner phase, respectively, forming a low-in-high-viscosity droplet in the upstream of the channel. The flow rate ratios of the low-viscosity phase to the high-viscosity phase are 3:1. The fluids in the injection syringe were driven through the Teflon capillary into the chip by the injection pump. When the low-in-high-viscosity droplet moved into the edge of the hydrophobic section, the high-viscosity lubricating film around the low viscous droplet broke up, forming a high-in-low viscous droplet in the downstream of the microchannel. Then the high-in-low sodium alginate droplets were collected in a petri-dish filled with 2 wt% calcium chloride solution. In general, the microfluidic chip can produce about 120 droplets per minute at 1440 mPa·s.

### 2.4. Drug Loading and Controlled Release

The prepared calcium alginates with nano–Fe_3_O_4_ microspheres were immersed in 1 wt% Blue-O (used as a model drug; Aladdin Co., Ltd., Shanghai, China) aqueous solution to load model drug in the hydrogel microspheres. A microcapillary tube was used as model blood vessels. Drug loaded microspheres were injected into the tube to simulate the situation where drugs flow in blood vessels. A magnet was used to control the movement of drug-loaded microspheres in the tube. A 45 °C hot water bag was placed on the tube to trigger the drug release of the microspheres at the designed place.

## 3. Results and Discussions

### 3.1. Effect of Flow Rates on Flowing Condition

**Figure 2:**
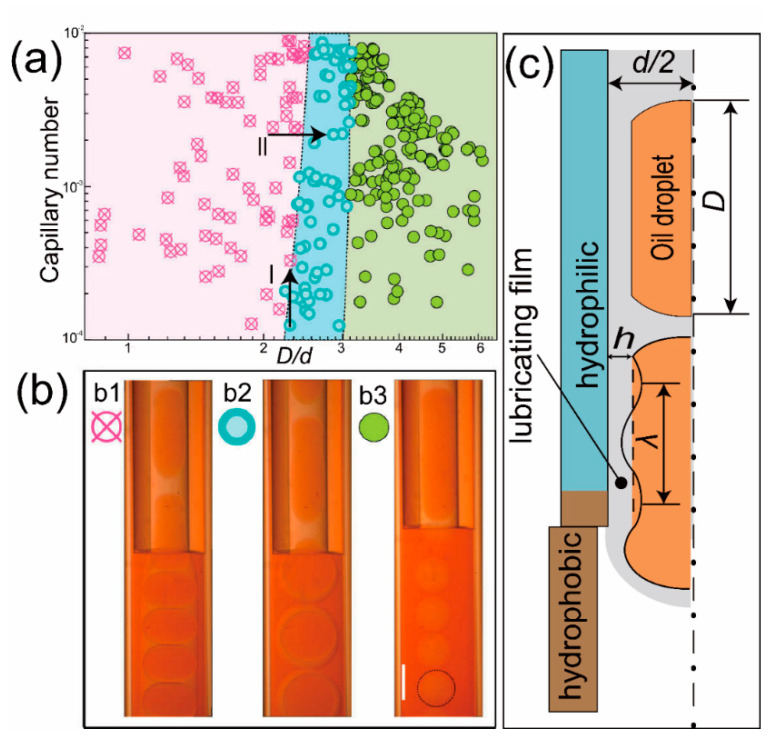
High-viscosity microspheres obtain by the phase-inversion method. (**a**) Log–log phase diagram of droplets inside the microfluidic chip. (**b1**) Low-in-high-viscosity droplets go through the orifice; (**b2**) Every two low-in-high-viscosity droplets break at the orifice every and form double emulsion droplets; (**b3**) Every low-in-high droplets break at the orifice and form single emulsion droplets. The scale bar is 500 μm; (**c**) Schematic illustration of the formation of high-viscosity droplets due to the breakup of the lubricating film.

Using the phase-inversion method, we could obtain monodispersed high-viscosity microspheres. However, the flowing condition of droplets in microfluidic chip varies when flow rates change. In this work, we gradually increased the flow rates, while keeping the flow rate ratio as constant and found that the phase diagram of the flowing condition can be divided into three different regions, as shown in Figure 2a. The phase-inversion phenomenon only occurred at low flow rates region. When the flow rates are larger than a critical value, phase-inversion could not happen. The flowing condition of droplets inside the microchannel exhibits different characteristics, as shown in Figure 2b.

It is known that in a co-flow microfluidic chip, the droplet size can be tuned by changing the flow rate ratio. If we gradually change the flow rate ratio to change the droplet size in the upstream, we can get the different value of *D/d*, while *D* is the length of the droplet (in orange color, as shown in Figure 2) in the upstream microchannel and *d* is the diameter of orifice. It can be found in Figure 2a that when *D/d* is smaller than 2.2, low-in-high-viscosity droplets go through the orifice, and phase-inversion phenomenon did not happen. We call this phenomenon “Go Through mode” (GTM). If 2.2 < *D/d* < 3, the first droplet goes through the orifice, but the flowing speed decrease when it goes into a larger space form the orifice. Accordingly, the second droplet breaks the high-viscosity lubricating film, forming a low-in-high-in-low double emulsion droplet. We call this phenomenon “Double Emulsion mode” (DEM). When *D/d* > 3, every droplet intends to break the lubricating film, forming high-viscosity microspheres one-by-one. We call this phenomenon “Single Emulsion mode” (SEM).

The reason why phase-inversion happens has been explained by the interactions between the attractive van der Waals force and the Laplace pressure of the curved droplet profile [59]. When a low-in-high-viscosity droplet moves into the hydrophobic channel, the long rang hydrophobic van der Waals force tends to pull the low viscous oil droplet onto the hydrophobic surface at the end of the orifice, as illustrated in Figure 2c. However, adhesion only occurs when the van der Waals force is equal to or larger than the Laplace pressure. The van der Waals force is given by the lubricating film thickness *h*, *P_van_* = A/(6π*h*^3^), where A is the Hamaker constant. The lubricating film is related to the capillary number according to the Bretherton equation, h/d~Ca^2/3^ [60]. The Laplace pressure at a curved interface can be calculated by *P*_s_ = *γ*/*r*_c_, where *r*_c_ is the radius of the curvature, which for a sinusoidally varying interface of wavelength *λ* and amplitude a is approximately *r*_c_ ~ *λ*^2^/a [59]. In our experiment, the wave amplitude *a* is approximately *h*, and the wavelength *λ* is linear to the length of the droplet *D*, which indicates that the Laplace pressure can be calculated by *P*_s_ = *γa*/*λ*^2^, as sketched by Figure 2c. At the adhesion moment, van der Waals force equals to the Laplace pressure, *P_van_ = P_s_*. For route I shown in Figure 2a, if we increase the *Ca* while keeping *D/d* constant, the lubricating film thickness *h* increases according to the Bretherton equation, leading to the decrease of *P_van_*, which means that the lubricating film can not be broken. For route II, if we increase the *D/d* while keeping the *Ca* constant, the wavelength *λ* increases due to the long droplet, leading to the decrease of *P_s_*, which means that the lubricating film is easy to break. The explanation fits well with the phase diagram shown in Figure 2a.

### 3.2. Control of the Size of Microspheres

**Figure 3:**
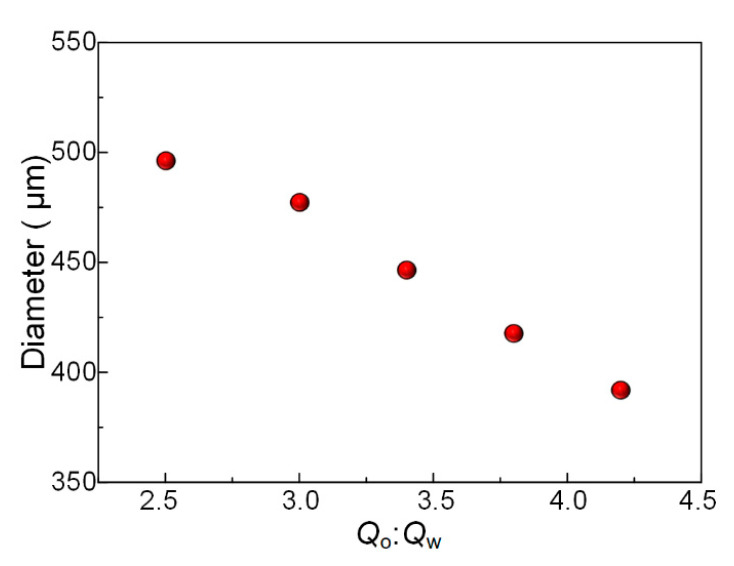
Control of the size of microspheres.

The narrow size distribution of microspheres prepared from the microfluidic chip is one of the main advantages of microfluidic technology. In this work, microspheres prepared using phase-inversion technology also have this advantage. We gradually increase the flow rate of low viscous fluid while keeping the high-viscosity fluid flow rate as 1.0 mL/h. Then we collected the microspheres prepared at different flow rate combinations and then measured their diameters. It can be found in Figure 3 that the diameter of the microspheres prepared from phase-inversion method can also be tuned through changing the flow rate ratio. The sizes of the high-viscosity microspheres were determined by the length of the high-viscosity fluid between two adjacent low viscous droplets in the upstream microchannel.

### 3.3. Structures of Microspheres

**Figure 4:**
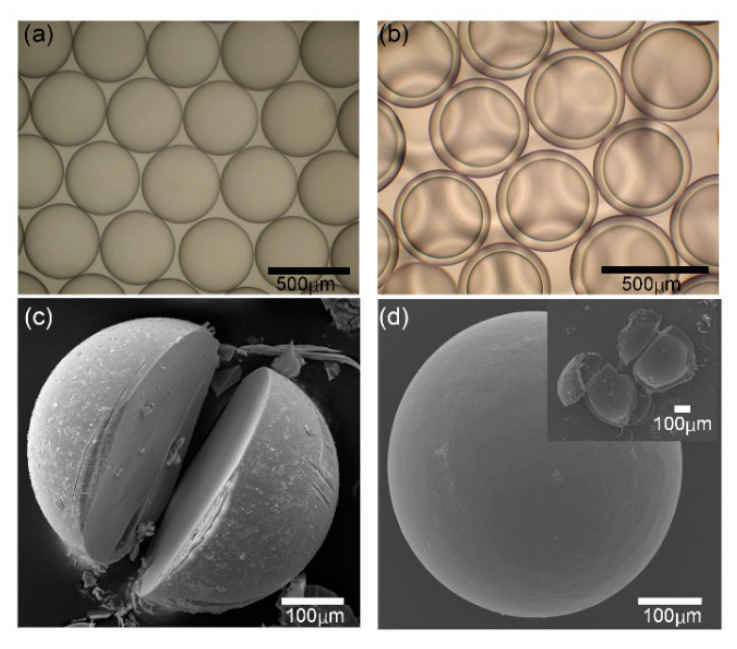
Calcium alginate microspheres prepared from the phase-inversion method. (**a**) Monodispersed microspheres prepared from SEM; (**b**) Monodispersed microspheres with core-shell structure prepared from DEM; (**c**) SEM image of solid microspheres; (**d**) SEM image of the core-shell microsphere and the inset shows its hollow structure.

We collected microspheres at different flow rate combinations and observed them in a microscope to evaluate the size distribution and degree of sphericity. First, we prepared microspheres in “SEM” and collect them in petri dish fulfilled with calcium chloride solution. The microspheres prepared from phase-inversion method have uniform size and good sphericity, as shown in Figure 4a. In addition, we collected microspheres in “DEM” and the double emulsion droplets also have narrow size distribution and good sphericity as shown in Figure 4b. Narrow size distribution is due to the accuracy control of fluids inside the microfluidic chip, while the good sphericity attributes to the larger surface tension compared with the gravity force in the microscale. Then we washed the microspheres using ethanol to remove the oil phase around the microspheres and then observed them in a scanning electron microscope. It can be found that the solid microsphere prepared from the phase-inversion method has a dense surface and also have a solid cross-section, as shown in Figure 4c. The microspheres prepared from “DEM” have the structures of core-shell or hollow inner, as shown in Figure 4d.

### 3.4. Application in Controlled-Drug-Release

**Figure 5:**
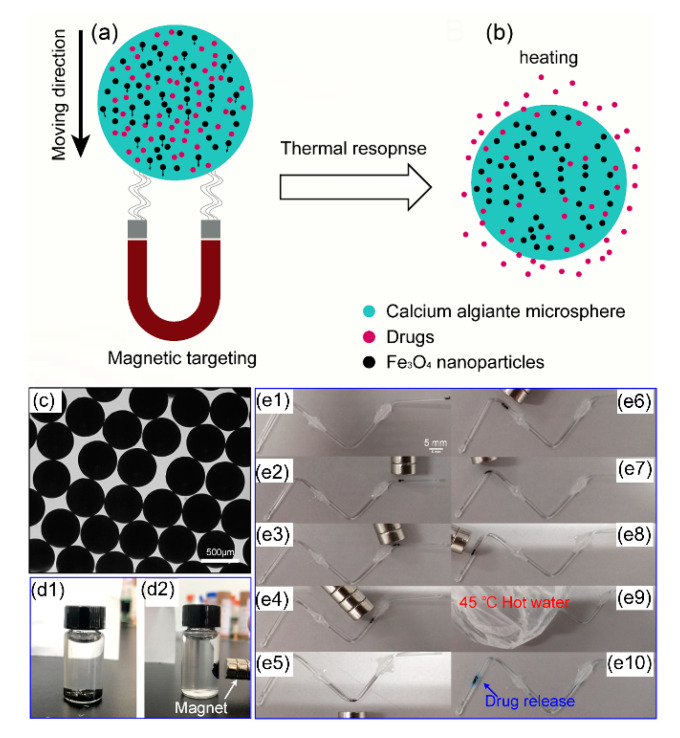
Application of magnet controlled calcium alginate microspheres in drug release experiment in a model vessel. (**a**) Moving mechanism of the magnetic calcium alginate microsphere; (**b**)The increasing drug release rate promoted by the thermal response of calcium alginate microsphere; (**c**) Monodispersed microspheres with Fe_3_O_4_ nanoparticles; (**d1**) Microspheres with Fe_3_O_4_ nanoparticles sink in water; (**d2**) Microspheres with Fe_3_O_4_ nanoparticles could be attracted by a magnet; (**e1**–**e8**) Microspheres with Fe_3_O_4_ nanoparticles can be guided to move by the magnet in a capillary tube; (**e9**) The microspheres were treated by a hot water bag to promote drug release; (**e10**) Blue-O in the microspheres were released after hot compress treatment.

Magnetic calcium alginate microsphere can be moved by an external magnetic field and controlled to release drugs by heating, as shown in Figure 5a,b. Magnet-controlled microspheres were prepared using the phase-inversion method and had a uniform size distribution as shown in Figure 5c. The microspheres were opaque because the nanoparticles inside the hydrogel blocked light transmission. The microspheres could be attracted by the magnet as shown in Figure 5d. Thus, we considered using the magnet to control the movement of microspheres. Here, we report a drug release experiment in a model vessel to show its excellent magnet-guided and thermo-controlled-release ability as drug delivery microcapsules. The model blood vessel was fulfilled with PBS solution and the microspheres were injected into the vessel from the right end. Then we used a magnet outside the vessel to control the movement of microspheres, as shown in Figure 5(e1–e8). When the microspheres reached the targeted place, a 45 °C hot water bag was placed on the tube to heat the microspheres, as shown in Figure 5(e9). After about 10 min, the model drug (Blue-O) was released from the microspheres as shown in Figure 5(e10), because calcium alginate was thermos-sensitive and the hydrogel structure would expand at a 45 °C environment. There are two reasons for the increased rate of drug release in a higher temperature environment. One is the increased rates of molecular movement with increasing temperature, and the other is that the sizes of pores in the inner gel network structure will increase, attributing to more easily release of the drug. These results indicate that the magnet controlled microspheres have great potentials in targeted drug delivery and precision medical treatment. The drug-delivered microspheres fulfilled with drugs could be injected into human blood vessels, and a magnet was placed at the lesion location to attract microspheres focusing here. Then a hot compress treatment could realize the controlled release at the targeted location.

## 4. Conclusions

In this work, we report a droplet-based bioprinting method for making high-viscosity calcium alginate microspheres using phase-inversion technology in a microfluidic bioprinting nozzle. High-viscosity alginate droplets can be prepared due to the breakup of lubricating film, forming monodispersed calcium alginate microspheres in the downstream microchannel. Different flowing conditions can be achieved by changing the flow rates, obtaining single and double emulsion droplets. Microspheres with structures of solid or core-shell can be prepared using the same method, only by changing the flow rates of fluid. Calcium alginate microspheres with nano–Fe_3_O_4_ particles made by this method have great potentials in magnet-guided and thermo-controlled drug-release, which are beneficial to the cell proliferation. This method is capable of making microspheres from high-viscosity bioink, which may have great potentials in tissue engineering, artificial organ and drug delivery et al.
